# Combining Multiple Assays Improves Detection and Serotyping of Foot-and-Mouth Disease Virus. A Practical Example with Field Samples from East Africa

**DOI:** 10.3390/v13081583

**Published:** 2021-08-10

**Authors:** Efrem Alessandro Foglia, Tiziana Lembo, Rudovick Kazwala, Divine Ekwem, Gabriel Shirima, Santina Grazioli, Emiliana Brocchi, Giulia Pezzoni

**Affiliations:** 1 Istituto Zooprofilattico Sperimentale della Lombardia e dell’Emilia Romagna (IZSLER), 25124 Brescia, Italy; santina.grazioli@izsler.it (S.G.); emiliana.brocchi@gmail.com (E.B.); giulia.pezzoni@izsler.it (G.P.); 2 The Boyd Orr Centre for Population and Ecosystem Health, Institute of Biodiversity, Animal Health & Comparative Medicine, University of Glasgow, Glasgow G12 8QQ, UK; tiziana.lembo@glasgow.ac.uk (T.L.); divine.ekwem@glasgow.ac.uk (D.E.); 3 The Nelson Mandela African Institution of Science and Technology, Arusha 23306, Tanzania; kazwala@gmail.com (R.K.); gabriel.shirima@nm-aist.ac.tz (G.S.)

**Keywords:** FMDV, detection assays, serotyping assays, comparison, field samples

## Abstract

Multiple serotypes and topotypes of foot-and-mouth disease virus (FMDV) circulate in endemic areas, posing considerable impacts locally. In addition, introductions into new areas are of great concern. Indeed, in recent years, multiple FMDV outbreaks, caused by topotypes that have escaped from their original areas, have been recorded in various parts of the world. In both cases, rapid and accurate diagnosis, including the identification of the serotype and topotype causing the given outbreaks, plays an important role in the implementation of the most effective and appropriate measures to control the spread of the disease. In the present study, we describe the performance of a range of diagnostic and typing tools for FMDV on a panel of vesicular samples collected in northern Tanzania (East Africa, EA) during 2012–2018. Specifically, we tested these samples with a real-time RT-PCR targeting 3D sequence for pan-FMDV detection; an FMDV monoclonal antibody-based antigen (Ag) detection and serotyping ELISA kit; virus isolation (VI) on LFBKαVβ6 cell line; and a panel of four topotype-specific real-time RT-PCRs, specifically tailored for circulating strains in EA. The 3D real-time RT-PCR showed the highest diagnostic sensitivity, but it lacked typing capacity. Ag-ELISA detected and typed FMDV in 71% of sample homogenates, while VI combined with Ag-ELISA for typing showed an efficiency of 82%. The panel of topotype-specific real-time RT-PCRs identified and typed FMDV in 93% of samples. However, the SAT1 real-time RT-PCR had the highest (20%) failure rate. Briefly, topotype-specific real-time RT-PCRs had the highest serotyping capacity for EA FMDVs, although four assays were required, while the Ag-ELISA, which was less sensitive, was the most user-friendly, hence suitable for any laboratory level. In conclusion, when the four compared tests were used in combination, both the diagnostic and serotyping performances approached 100%.

## 1. Introduction

Foot-and-mouth disease (FMD) is a serious transboundary infectious disease of livestock, which leads to considerable socio-economic impacts [1,2]. The aetiological agent of the disease, the FMD virus (FMDV), belongs to the genus *Aphtovirus*, family *Picornaviridae*, and exists as seven serotypes (O, A, C, Asia1, and Southern African Territory [SAT] 1–3) based on antigenic and immunological properties [3,4,5]. Moreover, because of its small single-stranded RNA genome, FMDV is subject to a constant evolutionary pressure, which results in the continuous emergence of new variants [6,7].

Currently, FMDV mostly circulates in the African and Asian continents, with occasional recurrence in South America. Sub-Saharan Africa, the Arabian Peninsula, the Middle East, India, and almost all of Far East and South-East Asia still experience endemic circulation of FMD viruses [8]. Depending on the characteristics of the different viruses circulating in these endemic areas, seven different viral pools have been identified, each persisting in a specific geographic region and comprising variants (alias topotypes) belonging to multiple (from three to five) serotypes. Genetically and antigenically distinct topotypes tend to occur within a defined regional virus pool, likely reflecting some degree of either ecological isolation or adaptation. Therefore, tailored diagnostics (as well as vaccines and control strategies) may be necessary to correctly capture this diversity [9].

When an outbreak is suspected based on clinical presentation, rapid and accurate diagnosis is essential to inform outbreak management decisions and contain further spread [10]. Identification of serotype, topotype, and variant, combined with phylogenetic analyses, is essential to determine the origin of the outbreak and monitor onward dissemination to other areas. Fast and reliable characterisation of circulating viruses critically enables epidemiological analyses and vaccine choice [10].

Recent years have seen many diagnostic advances towards faster detection and serotyping, in order to compensate for some weaknesses of traditional methods [11], including virus isolation (VI) [11,12], Ag-detection ELISA (Ag-ELISA) [11,13], and pan-reactive real-time RT-PCRs [14,15,16,17]. For example, VI is time consuming, can detect only viable virus, and requires an accompanying test for the identification of the virus; Ag-ELISA has low sensitivity and might fail to detect virus on poor field samples; and no serotyping is possible using pan real-time RT-PCR. Therefore, research on FMD diagnostics focuses on rapid molecular assays specific for each serotype, which can both confirm the presence of virus and type it in a single reaction [18,19]. Unfortunately, because of the high inherent genetic variability of serotypes, no such test has yet been developed. However, by combining information available about FMDV topotypes of each pool, precise panels of real-time reactions could be tailored to the needs of a specific area [20,21,22,23,24].

In this study, we compared commonly used methods for FMDV detection and typing using field samples collected in East Africa (EA) from cattle displaying clinical signs consistent with FMD. Type O, A, SAT1, and SAT2 viruses belonging to pool four circulate in this region, and the most recent topotypes affecting this area were A/AFRICA/G-I in 2013, O/EA2 and SAT1/I (NWZ) in 2014, and SAT2/IV in 2012 [25]. The assays included in our comparison were: the real-time RT-PCR targeted on 3D sequence for pan-FMDV detection [14]; a monoclonal antibodies (MAbs)-based FMDV antigen detection and serotyping ELISA (Ag-ELISA) [13]; VI on a continuous porcine cell line [11,26], combined with the above Ag-ELISA; and a panel of four real-time RT-PCRs tailored for the topotypes circulating in the EA region [23]. We report the results of assay comparison, and highlight critical issues emerging during the diagnostic and characterisation process, with particular emphasis on the time required to serotype samples. We discuss factors that might affect speed, for example, the number of reactions required, the quality and quantity of samples, and the presence of contaminants.

## 2. Materials and Methods

### 2.1. Samples Collection and Preparation

In total, 127 epithelial tissues were collected between 2012 and 2018 in Serengeti District (northern Tanzania) from the gums or feet of animals with FMD clinical signs, and prepared following recommended procedures [12]. Briefly, in order to obtain sterile tissue homogenates, tissue samples up to 1 g were grinded with quartz powder, resuspended in 5 mL of MEM (added with L-glutamine 2 mM, penicillin 75 μg/mL, and streptomycin 100 μg/mL), centrifuged 30 min at 12,000× *g*, and filtered with 0.45 μm syringe filters.

### 2.2. Viral RNA Extraction

Viral RNA was extracted from 200 μL of original homogenates or isolates (cell culture supernatants) using MagMAX^TM^ CORE Nucleic Acid Purification Kit (Applied Biosystems by Thermo Fisher Scientific, Waltham, MA, USA) on KingFisher Duo Prime System (Thermo Fisher Scientific, Waltham, MA, USA), as recommended by the manufacturers. The viral RNA, eluted in 60 μL of specific buffer, was stored at −70 °C until used in the experiments.

### 2.3. Real-Time RT-PCR Targeted on 3D Sequence and Topotypes Specific Real-Time RT-PCRs Tailored for EA

The real-time RT-PCR targeted on 3D sequence [14] and topotype specific real time RT-PCRs [23] were performed with SuperScript^TM^ III Platinum^TM^ One-Step qRT-PCR System (Invitrogen by Life Technologies, Thermo Fisher Scientific, Waltham, MA, USA), as recommended by the manufacturers, starting from 5 μL of eluted RNA. For all the 5 reactions, a unique thermal profile was carried out, inclusive of an RT step (30 min at 60 °C and 10 min at 95 °C) and 50 cycles of amplification (15 s at 95 °C and 1 min at 60 °C each).

### 2.4. Monoclonal Antibodies (MAbs)-Based ELISA for FMDV Antigen Detection and Serotyping (Ag-ELISA)

Detection and serotyping of FMDV by Ag-ELISA was carried out using an updated version of the FMDV Antigen Detection and Serotyping ELISA Kit (IZSLER, Brescia, Italy and TPI, Pirbright, UK) [13], including detection and typing of serotypes SAT1 and SAT2, in addition to O, A, C, and Asia 1. Manufacturers’ instructions were followed. Briefly, 250 μL of original tissue homogenates or cell culture supernatants were diluted 1:2 in the specific dilution buffer and incubated for 1 h at room temperature into one row of the ELISA plate, 50 μL per well, pre-coated with the battery of selected type-specific capture MAbs and one additional pan-FMDV MAb. The same pan-FMDV MAb conjugated to peroxidase (Conjugate A) was used to complete the detection and typing of serotypes O, A, C, and Asia 1, while a mix (Conjugate B) comprising one SAT1, one SAT 2, and one cross-reactive MAbs was used for the detection and typing of SAT 1 and SAT 2 serotypes. The two Conjugates were incubated for 1 h at room temperature, after which the reaction was developed using TMB (3,3′,5,5′-tetrametilbenzidina) as substrate. After the addition of a stop buffer (H_2_SO_4_ 0.6 N), optical density was measured at 450 nm using an ELISA reader. Washes between each step were required.

### 2.5. Virus Isolation on a Continuous Porcine Kidney Cell Line

Virus isolation was carried out using a continuous porcine kidney cell line, LFBKαVβ6, kindly provided by Luis L. Rodriguez (Plum Island Animal Disease Centre, Agricultural Research Service, USDA, Plum Island, NY, USA). This cell line is specifically engineered to constitutively express bovine αVβ6 integrin [26], which increases susceptibility to FMDV and speeds up virus replication. Infections were carried out as previously described [11,12]. Briefly, 500 μL of homogenates were inoculated with 500 μL of MEM (added with l-glutamine 2 mM, penicillin 75 μg/mL, and streptomycin 100 μg/mL) on cell monolayer (70–80% confluence) in a flask with 25 cm^2^ surface area and incubated at 37 °C for 1 h. After incubation, the inocula were aspirated and wasted, and the flasks were incubated at 37 °C for up to 48 h with 10 mL of fresh MEM (added with l-glutamine 2 mM, penicillin 75 μg/mL, and streptomycin 100 μg/mL) with foetal bovine serum 3%. Isolates were harvested when the inoculated cells showed a complete cytopathic effect and the virus type was confirmed by the Ag-ELISA, as described above. If following sub-cultures were needed, 1 mL of harvested supernatants was filtered with 0.45 μm syringe filters, and inoculated with 9 mL of fresh MEM (added with l-glutamine 2 mM, penicillin 75 μg/mL, and streptomycin 100 μg/mL) with foetal bovine serum 3% on cell monolayer (70–80% confluence) in a flask with 25 cm^2^ surface area and incubated at 37 °C for up to 48 h. If the third sub-culture does not show a cytopathic effect, the sample is considered as negative.

### 2.6. VP1 Sequencing

VP1 sequencing was conducted by Sanger techniques, as previously described [27]. Briefly, end-point RT-PCRs serotype-specific were performed with QIAGEN^®^ OneStep RT-PCR Kit (QIAGEN GmbH, Hilden, Germany) on RNA extracted from all culture supernatants positive to VI. Primers were those previously and exhaustively described [28]. RT-PCR products were run on a 2% agarose gel, and those of the expected size were purified from agarose using NucleoSpin Gel and PCR Clean-up kit (Macherey-Nagel GmbH & Co. KG, Düren, Germany). Purified amplicons were sequenced with Applied Biosystems 3500 XL Genetic Analyzer by an automated fluorescence-based technique, following the manufacturer’s instructions (Thermo Fisher Scientific, Waltham, MA, USA). Obtained fragments were assembled in a unique sequence for each sample using the software SeqMan Pro-Lasergene 10 Core Suit (DNASTAR, Madison, WI, USA).

## 3. Results

### 3.1. Pan FMDV Real-Time RT-PCR Targeted on 3D Gene

Using the 3D real-time RT-PCR [14], a total of 8 out of 127 sample homogenates yielded negative results, with 6 of them also remaining negative when analysed using the further detection and serotyping tests involved in this work (Table 1). This suggests that the latter were from FMDV uninfected cattle (interestingly, Bovine Viral Diarrhoea Virus was isolated from three of them), and that the test failed to detect FMDV on only two samples which were identified as positive by the other techniques included in the investigation, i.e., VI for one and topotype-specific real-time RT-PCRs for both of them (Table 1).

A total of 119 tissue homogenates, excluding the 6 negative and 2 untyped samples (the latter positive to the pan-reactive real-time RT-PCR only), were included in the evaluation of the typing performance of the assays, as described below. Even if VI alone is not a typing method, the virus enrichment obtained with this technique combined with Ag-ELISA performed on cell culture supernatants returned serotype level information.

### 3.2. Ag-ELISA

**Figure 1:**
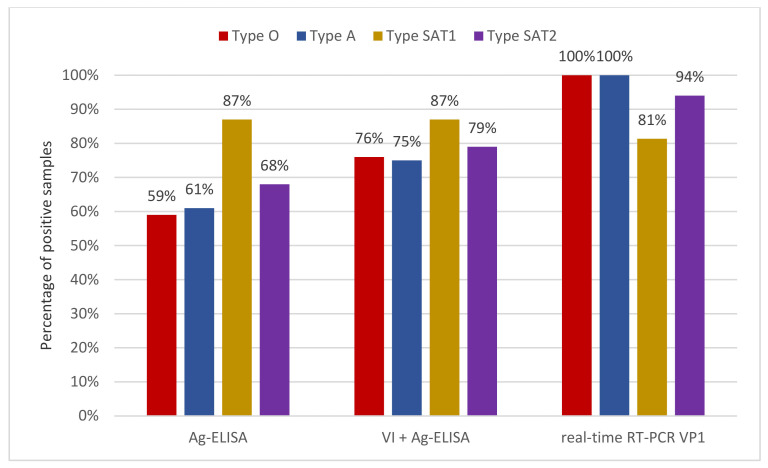
Serotyping performances of three assays, calculated as the proportion of samples correctly typed. From the left, Ag-ELISA on tissue homogenates (Ag-ELISA); VI and subsequent Ag-ELISA (VI+ following Ag-ELISA); and topotype-specific real-time RT-PCRs targeted on VP1 (real-time RT-PCR VP1). Results are shown by serotype: in red type O, in blue type A, in yellow type SAT1, and in purple type SAT2, according to the colour scheme proposed by by the World Reference Laboratory for Foot-and-Mouth Disease. Percentages are calculated based on the cumulative number of FMDV samples positive for each serotype (including the samples positive for two serotypes): 29 for type O, 44 for type A, 32 for type SAT1, and 19 for type SAT2. Topotype-specific real-time RT-PCRs showed the best sensitivity among assays, except for the SAT1-specific reaction, which had lower sensitivity.

The Ag-ELISA kit incorporating type-specific MAbs, together with a pan-FMDV antigen reaction [13], is the most user-friendly method for serotyping FMD viruses. It can be performed in less than three hours in laboratories with limited infrastructure. In this study, this assay was able to detect and type FMDV in 71% (85/119) of tissue homogenates (Figure 1). The lower detection rate of the Ag-ELISA is likely attributable to an intrinsic reduced analytical sensitivity of the test, compared to real-time RT-PCR. Concerning the detection frequency of various serotypes, the Ag-ELISA correctly recognised 87% of SAT1 samples (28/32), 68% of type SAT2 (13/19), 61% of type A (27/44), and 59% of type O (17/29) (Table 1 and Figure 1).

### 3.3. VI Combined with Ag-ELISA on Culture Supernatants

VI is the most time-consuming and laborious method among the compared assays. Using LFBKαVβ6 [26], 98 out of the 119 samples considered (82%) induced effective infection on the cell line after 24–48 h. The virus isolates were subsequently typed by Ag-ELISA (Table 1): 76% were type O (22/29), 75% type A (33/44), 87% SAT1 (28/32), and 79% SAT2 (15/19; Table 1 and Figure 1).

### 3.4. Real-Time RT-PCRs Tailored to Topotypes Circulating in EA

Finally, we included a panel of four real-time RT-PCRs in our comparison, tailored to the pool 4 topotypes circulating in Tanzania. In general, the performance of the reaction panel was high, approaching that observed for 3D-targeted real-time RT-PCR. It correctly identified 94% of positive samples (112/119; Table 1). The identified serotypes were subsequently confirmed by VP1 sequencing of the products of the serotype-specific end-point RT-PCRs, carried out on the corresponding isolates by Sanger technique [27]. The performance of the four real-time RT-PCRs varied depending on the topotype. Reactions tailored to O/EA2-4 and A/AFRICA/G-I detected all samples (28/28 and 41/41, respectively; Table 1 and Figure 1), and that tailored to SAT2/IV detected 94% of samples, i.e., only 1 out of 18 samples was missed (Figure 1 and Table 1). In contrast, only 81% (26/32) of SAT1 positive samples were detected by the real-time RT-PCR tailored to topotype SAT1/I (Table 1 and Figure 1). All negative samples were also tested ten-fold diluted, in order to exclude the presence of inhibitors. Interestingly, five samples resulted contemporarily positive to two different topotype-specific real-time RT-PCRs. Specifically, one sample type A showed low positivity for type SAT2 reaction (42.80 Ct value; Table S2), one sample SAT1 and two samples SAT2 were also weak positive to the reaction against type A (31.64, 33.33, and 37.21 Ct values, respectively; Table S2), and one sample type SAT2 was also positive for type O (41.13 Ct value; Table S2).

## 4. Discussion

In order to achieve a fast and consistent FMD diagnosis, many different variables may influence the analysis, such as the time needed to conduct the test, the goodness of the samples, and the quantity of the analyte.

Overall, the presented work showed that molecular assays for FMDV detection and/or serotyping performed better, compared to those based on Ag-ELISA or viral isolation. However, a number of caveats emerged that we discuss below.

Pan-reactive real-time RT-PCR was confirmed as the most sensitive test for FMDV detection, with a diagnostic sensitivity of 98% (119/121; Table 1). Considering the limited amount of time required for test completion i.e., around 3 h, this assay is optimal for screening. An important shortcoming, however, is that it cannot distinguish between serotypes.

Although Ag-ELISA is characterised by lower sensitivity compared to other assays, in our study it was able to detect and type six samples (3 type A, 2 SAT1, and 1 SAT2), which were undetected by VI, likely because of poor or lost virus viability in these specimens.

Two samples were missed by both 3D real-time RT-PCR and Ag-ELISA, but were properly characterised by the topotype-specific real-time RT-PCRs, one as type O and another one as type A (Table 1). While the negative result in Ag-ELISA may be due to low sensitivity, the results of the pan-FMDV real-time were unexpected. These findings were confirmed using another pan-FMDV real-time RT-PCR targeting 5′ UTR [17] that also yielded negative results. Testing of a ten-fold dilution of these samples did not change the results, thus excluding inhibitors’ presence in the reaction. The type O-positive sample could not be isolated on LFBKαVβ6 cells (Table 1), whilst the type A-positive sample turned out positive also by 3D real-time RT-PCR after isolation on the cell line (Table 1). A low virus concentration in both original samples might explain the negative results of the pan-molecular assay. Indeed, Ct values generated by O-specific and A-specific real-time RT-PCRs on RNA extracted by original samples were very high (39.03/45.77 for O/EA2-4 real-time RT-PCR on the first sample and 47.38 for A/AFRICA/G-I real-time RT-PCR on the second), confirming low virus concentration in those samples.

Conversely, a low concentration of virus in the samples missed by the SAT1/I (NWZ) real-time RT-PCR might not explain the failure of this assay. The six samples, negative by the topotype-specific real-time RT-PCR, were positive by 3D real-time RT-PCR, with Ct values between 10.26 and 20.87. These results were comparable to those obtained on the other SAT1 samples, which had Ct values for 3D ranging between 10.12 and 22.25, confirming that the samples had equivalent virus amounts (Table S1). Moreover, in samples positive by both assays, Ct values of pan-3D and SAT1-specific real-time RT-PCRs were comparable, suggesting similar analytical performance of the two assays (Table S1). Finally, VP1 sequences generated from SAT1 isolates were aligned and analysed to find mutations in the negative samples that would explain negative real-time RT-PCR outcomes. However, no specific mutations were detected in the region targeted by the reaction. Equally unexpectedly, these six negative samples were correctly detected and typed as SAT1, even by the less sensitive Ag-ELISA technique.

Two samples were positive only in 3D real-time RT-PCR and therefore remained untyped (Table S3). A possible reason for this is that the samples had low virus concentration and/or contained largely degraded viral RNA or unviable virus. Persistently negative results by real-time RT-PCRs specific to topotypes circulating in East Africa suggest that further topotypes might be circulating in the area. If this were the case, new reliable tests that are able to identify all FMDV variants belonging to a specific serotype with high sensitivity, accuracy, and speed would be necessary.

All of these considerations proved that many different issues may interfere with a fast and reliable detection/serotyping of FMDV in field samples, highlighting the importance of combining various assays together, in order to obtain a diagnosis that is as accurate as possible.

Among the 119 samples typed, five showed positivity to two different serotypes (Table 1 and Table S2). This finding is worthy of special consideration. FMD outbreaks are usually described as epidemic waves, each characterised by the circulation of one dominant FMDV serotype. However, these different waves may be overlaid, and individual animals may be infected with distinct viruses simultaneously [25,28,29,30]. In our study, traditional assays were not able to detect two serotypes in a single sample. Specifically, all five samples were confirmed to be positive by 3D real-time RT-PCR, and were typed as a specific serotype (namely one A, one SAT1, and three SAT2) by Ag-ELISA, and VI combined with Ag-ELISA (Table 1 and Table S2). However, topotype-specific real-time RT-PCRs carried out on original tissue homogenates showed that in those samples, two different serotypes were detectable contemporarily: three A/SAT2, one A/SAT1, and one O/SAT2. In general, amongst the five samples where two viruses of different serotypes were present, only the virus type which was most abundant (according to lower Ct values recorded by molecular assays) was revealed by the Ag-ELISA on tissue homogenates and/or isolated in cells, with the exception of type A which apparently showed less fitness when competing with other serotypes for growth in LFBKαVβ6. Interestingly, the three samples positive for A and SAT2 types were collected from the same farm during a single outbreak. In these samples, although the Ct values of the type-specific RT-PCRs suggested that type A was more represented, VI in LFBKαVβ6 cells selected and favoured the growth of the SAT2 virus from two of the three cattle. Type A virus emerged from the third one, in line with a positive result in Ag-ELISA and the very low Ct values observed in the type-specific molecular tests (Table 1 and Table S2). These double infections would have remained undisclosed without the use of the topotype-specific real-time RT-PCRs.

In conclusion, this work showed that (1) the 3D targeted real-time RT-PCR alone is the most reliable test for FMDV detection when serotyping is not necessary; (2) the MAbs-based Ag-ELISA kit is the most user-friendly assay with the advantage of typing capability, thus it is suitable for any laboratory level despite its lower sensitivity; (3) VI is the least practical, and its performance is sub-optimal but is useful to enrich virus for subsequent typing, including antigenic profiling, and, eventually, vaccine matching assays; and (4) topotype-specific real-time RT-PCRs are the most effective assays for simultaneous detection and serotyping, despite the need for previous information about circulating topotypes and the requirement for multiple reactions. We demonstrated that, taken together, traditional assays (pan-FMDV real-time, VI, and ELISA) detected virus in almost all (99%; 120/121) cases and enabled serotyping of 86% (104/121) of positive samples. However, when combined with more recent molecular techniques, their overall performance improved, especially in terms of serotyping (typing 98%; 119/121). This confirms that the continuous development of new and reliable diagnostic methods is necessary for improving detection and enabling the characterisation of FMDV in field samples.

## Figures and Tables

**Table 1:** Summary of the results obtained using four assays examined in this study. A real-time RT-PCR targeted on 3D sequence for pan-FMDV detection (real-time RT-PCR 3D); the monoclonal antibodies-based FMDV antigen detection and serotyping ELISA kit (Ag-ELISA); virus isolation on LFBKαVβ6 cell line combined with the above Ag-ELISA kit (VI+Ag-ELISA); and a panel of four real-time RT-PCRs tailored for the topotypes circulating in the East Africa region (real-time RT-PCR VP1). Colours refer to the four serotypes following the scheme proposed by the World Reference Laboratory for Foot-and-Mouth Disease: in red type O, in blue type A, in yellow type SAT1, and in purple type SAT2. *, samples from which two serotypes were detected contemporarily using the topotype-specific real-time RT-PCRs.3.2. Detection and Serotyping Methods.

		Typing Assays				
	Real-Time RT-PCR 3D	Ag-ELISA	VI (+Ag-ELISA)	Real-Time RT-PCR VP1	Samples Number	Percentage
**Type O**	POS	POS	POS	POS	17	**61%**
	POS	NEG	POS	POS	5	**18%**
	POS	NEG	NEG	POS	5	**18%**
	NEG	NEG	NEG	POS	1	**3%**
**Tot Pos**	**27**	**17**	**22**	**28**	**28**	
**Type A**	POS	POS	POS	POS	23 + 1 *	**59%**
	POS	NEG	POS	POS	8	**20%**
	POS	NEG	NEG	POS	5	**12%**
	POS	POS	NEG	POS	3	**7%**
	NEG	NEG	POS	POS	1	**2%**
**Tot Pos**	**40**	**27**	**33**	**41**	**41**	
**Type SAT1**	POS	POS	POS	POS	19 + 1 *	**63%**
	POS	NEG	POS	POS	2	**6%**
	POS	NEG	NEG	POS	2	**6%**
	POS	POS	NEG	POS	2	**6%**
	POS	POS	POS	NEG	6	**19%**
**Tot Pos**	**32**	**28**	**28**	**26**	**32**	
**Type SAT2**	POS	POS	POS	POS	11 + 1 *	**67%**
	POS	NEG	POS	POS	1 + 2 *	**17%**
	POS	NEG	NEG	POS	2	**11%**
	POS	POS	NEG	NEG	1	**5%**
**Tot Pos**	**18**	**13**	**15**	**17**	**18**	
**Serotyped Samples**					**119**	**94%**
**Unserotyped Samples (real-time RT-PCR 3D POS)**					**2**	**1%**
**Negative Samples**					**6**	**5%**
**Total Samples Tested**					**127**	

## Data Availability

Not applicable.

## Supplementary Materials

The following are available online at https://www.mdpi.com/1999-4915/13/8/1583/s1. Table S1: list of samples resulted positive to FMDV SAT1 with real-time RT-PCR results; Table S2: Ag-ELISA and real-time RT-PCR results for samples in which two serotypes were detected; Table S3: samples positive only by 3D real-time RT-PCR.
