# Supplementary material for: Combining Multiple Assays Improves Detection and Serotyping of Foot-and-Mouth Disease Virus. A Practical Example with Field Samples from East Africa
Source: Viruses. 2021 Aug 10;13(8):1583. doi: 10.3390/v13081583 (PMC8412026; doi:10.3390/v13081583)
Supplement: Supplementary file 1 [file viruses-13-01583-s001.zip › viruses-1314049-supplementary.pdf]

Supplementary Materials

Table S1. List of samples resulted positive to FMDV SAT1 with real-time RT-PCR results.

SAMPLE No	Ct values real-time RT-PCR 3D	Ct values real-time RT-PCR VP1
**392225**	30,04	28,72
**378140**	19,79	22,08
**378159/1**	15,99	20,21
**378177/1**	19,27	24,18
**378177/2**	17,02	19,75
**378194/1**	14,30	15,87
**378194/2**	16,77	18,64
**378199**	10,19	10,96
**378205/1**	15,18	16,91
**378205/2**	13,24	13,78
347782/1	19,48	nd
350434/1	19,61	nd
350434/2	20,87	nd
350434/3	10,26	nd
350434/4	17,27	nd
**350434/5**	17,33	19,87
**353693/2**	19,79	19,69
**353693/3**	13,51	15,63
**353693/4**	10,29	12,76
**353717/1**	17,34	23,53
353717/3	13,45	nd
**359649/1**	18,56	16,08
**359674/2**	15,82	17,05
**359674/3**	16,90	19,07
**359674/4**	10,12	10,06
**359674/5**	16,24	15,19
**359674/7**	22,25	25,18
**359708/1**	18,24	26,69
**359708/2**	17,45	23,92
**359708/4**	14,18	17,20
**359717**	16,93	20,83

This table lists all samples resulted positive to SAT1 (first column) and shows the Ct values of pan-FMDV real-time RT-PCR (second column) and topotype-specific real-time RT-PCR (third column). Samples not detected (nd) by the specific reaction are in italic. In these samples the pan-FMDV test yielded Ct values within the same range of all other samples, thus excluding low virus concentration as cause for failure. nd, not detected.

Table S2. Ag-ELISA and real-time RT-PCR results for samples in which two serotypes were detected.

SAMPLE No	Ag-ELISA	VI + Ag-ELISA	Ct values real-time RT-PCR 3D	Ct values real-time RT-PCR VP1	
**40783/1**	SAT2	SAT2	13,84	SAT2/IV 34.54	O/EA2-4 41.13
**211987/1**	NEG	SAT2	25,13	A/AFRICA/G-I 25.66	SAT2/IV 33.33
**211987/2**	NEG	SAT2	24,68	A/AFRICA/G-I 24.24	SAT2/IV 37.21
**211987/3**	A	A	7,83	A/AFRICA/G-I 7.26	SAT2/IV 42.80
**378194/1**	SAT1	SAT1	14,3	SAT1/I (NWZ) 15.87	A/AFRICA/G-I 31.64

This table includes all samples in which two serotypes were detected (first column), the results of Ag-ELISA testing (second column), the type of isolated virus (third column) and the Ct values of pan-FMDV real-time RT-PCR (fourth column) and of the topotype-specific real-time RT-PCRs showing a positive outcome (fifth and last column). Samples with consequential numbers (i.e. 211987/1, 2 and 3) were collected contemporarily in the same farm and according to type-specific real-time RT-PCRs all harbour viruses of serotype A and SAT2; however, even if all three samples displayed lower Ct for type A reaction, in the first two the type SAT2 virus was isolated on LFBK cell line, while the type A virus emerged in culture from the third sample, which was even more rich in this serotype according the real-time RT-PCR outcomes.

Table S3. Samples positive only by 3D real-time RT-PCR.

SAMPLE No	Ct values real-time RT-PCR 3D	Ct values real-time RT-PCR VP1	Type
**40767**	23.25	nd	nd
**347782/2**	34.94	nd	nd

This table lists the two samples positive (first column) only by real-time RT-PCR targeted on 3D (second column), with relevant Ct values. These samples could not be typed, neither by topotype-specific real-time RT-PCR (third column) nor by any other test (fourth column; nd: not detected).
